# Total 25(OH)D Concentration Moderates the Association between Caffeine Consumption and the Alkaline Phosphatase Level in Pregnant Women

**DOI:** 10.3390/nu14081616

**Published:** 2022-04-13

**Authors:** Keith T. S. Tung, Rosa S. Wong, Calvin K. M. Cheung, Jennifer K. Y. Ko, Bianca N. K. Chan, Albert Lee, Hung-Kwan So, Wilfred H. S. Wong, Wing-Cheong Leung, Patrick Ip

**Affiliations:** 1 Department of Paediatrics and Adolescent Medicine, The University of Hong Kong, Hong Kong SAR, China; keith-tung@connect.hku.hk (K.T.S.T.); rosawg@connect.hku.hk (R.S.W.); bbianca@hku.hk (B.N.K.C.); hkso@hku.hk (H.-K.S.); whswong@hku.hk (W.H.S.W.); 2 Department of Social Work and Social Administration, Sau Pao Centre on Ageing, Centre on Behavioral Health, The University of Hong Kong, Hong Kong SAR, China; 3 Department of Pharmacology and Pharmacy, The University of Hong Kong, Hong Kong SAR, China; 4 School of Public Health and Primary Care, The Chinese University of Hong Kong, Hong Kong SAR, China; calvincheung@cuhk.edu.hk (C.K.M.C.); alee@cuhk.edu.hk (A.L.); 5 Department of Obstetrics & Gynaecology, Queen Mary Hospital, Hong Kong SAR, China; jenjenko@gmail.com; 6 Department of Obstetrics & Gynaecology, Kwong Wah Hospital, Hong Kong SAR, China; leungwc@ha.org.hk

**Keywords:** pregnant women, caffeine consumption, alkaline phosphatase, food frequency questionnaire

## Abstract

The evidence as to whether caffeine consumption is beneficial or harmful to human health has been mixed. This study aimed to examine the effect of 25-hydroxyvitamin D (25(OH)D) concentration on the association between caffeine consumption and mineral metabolism in pregnant women. This is a cross-sectional study involving pregnant women at their 25th to 35th gestational week recruited at antenatal clinics in the period of July 2019 to December 2020. Peripheral blood samples were collected to determine their total 25(OH)D, albumin, alkaline phosphatase (ALP), calcium, phosphate, and ferritin level in serum. Questionnaires on demographics and dietary intake were also administered. Among 181 pregnant women recruited (Average age = 32.9 years), 50 (27.6%) of them were found to be vitamin D insufficient (25(OH)D concentration < 75 nmol/L), and 131 (72.4%) were vitamin D sufficient (25(OH)D concentration ≥ 75 nmol/L). Adjusted regression models identified an association between higher caffeine intake and lower ALP level only among vitamin D-sufficient pregnant women (β = −0.24, *p* = 0.006), but not in those with insufficient vitamin D (β = −0.02, *p* = 0.912). The findings provide new insights into 25(OH)D concentration as a potential modifier of the health effects of caffeine consumption during pregnancy.

## 1. Introduction

Caffeine is a psychostimulant that is widely found in food, beverage, and medicine. Its primary functions are to reduce physical fatigue and improve mental alertness [1]. While the consumption of caffeine-containing foods and beverages is popular worldwide, the health impact of caffeine consumption during pregnancy has been a matter of debate [2]. Guidelines developed by the European Food Safety Authority and the American Institute of Medicine recommend pregnant women consume no more than 200 mg of caffeine each day [3]. Likewise, a previous systematic review indicated no safe amount of caffeine consumption during pregnancy [4]. Nonetheless, a cohort study found that over one third of the pregnant women continued to consume caffeine-containing beverages throughout pregnancy [2].

The evidence as to whether caffeine consumption is beneficial or harmful to human health has been mixed. While some epidemiological studies reported the health benefits of caffeine consumption, such as reduced risks of Alzheimer’s disease and type 2 diabetes [5], others have found that its effects on bone metabolism and cardiovascular health are potentially harmful [1]. Pregnant women, in particular, may have stronger physiological responses to caffeine intake, as the half-life of caffeine is prolonged 1.5 to 3.5 times during pregnancy due to the decreased activity of cytochromes P450 in the liver [6]. These responses may in turn impair foetal growth and development [4].

In addition, some evidence suggests that caffeine consumption is associated with inhibition of intestinal calcium absorption and increased urinary excretion of calcium and magnesium [7]. Caffeine consumption may also affect other biochemical markers, such as albumin, alkaline phosphatase (ALP), phosphate, and ferritin. Specifically, ALP is an enzyme circulating in the bloodstream and is found in various body parts including the liver, bones, and placenta [8]. Changes in ALP levels can affect liver and bone health [9,10]. However, a recent systematic review found no adverse health effects of caffeine consumption among healthy pregnant women [11]. These inconsistent associations may be due to the methodological variations in the measurement of caffeine consumption and confounding adjustment. For example, vitamin D is a steroid hormone that plays an important role in regulating bone mineralisation, calcium absorption, and immune responses activation [12]. Preliminary evidence shows that the health effect of caffeine could vary by vitamin D receptor genotypes [13]. More investigations are thus needed to determine whether the effect of caffeine consumption may depend on other physiological conditions including those relating to vitamin D.

Achieving adequate vitamin D levels is particularly important for pregnant women since their vitamin D content can be transferred to their foetuses [14]. While it is recommended for pregnant women to take a routine daily vitamin D supplementation of 600 IU during pregnancy [15], the prevalence of vitamin D insufficiency remains high in the Chinese population [14]. A previous study on Chinese pregnant women found that 18% of pregnant women in Hong Kong and 40% in Beijing were found to be vitamin D deficient [14]. Considering the important role of vitamin D in the health of pregnant women, the aim of this study is to investigate the association between caffeine intake and mineral metabolism among pregnant women of different vitamin D statuses in Hong Kong.

## 2. Method

### 2.1. Study Design and Participants

This cross-sectional study was conducted in the period of July 2019 to December 2020. A total of 181 healthy pregnant women were randomly approached and recruited by trained research staff during their antenatal clinic visits in local public hospitals in Hong Kong at their 25th to 35th gestational week. Pregnant women who (i) were not capable of speaking or reading Chinese; (ii) had any chronic medical problems including metabolic bone disease, calcium disorder, thyroid disease and (iii) had been subscripted with vitamin D supplementation as treatment, were excluded from this study. Upon informed consent, the women were asked to complete a questionnaire on their demographics and dietary intake. Peripheral blood samples were collected from them by a well-trained phlebotomist. An incentive of a 100 HKD supermarket voucher was given to the participants upon the completion of the study assessment.

### 2.2. Measures

#### 2.2.1. Biochemical Outcomes

Serum was extracted from the collected peripheral blood samples to determine the vitamin D status of the pregnant women by measuring the total 25(OH)D concentration. The liquid chromatography-tandem mass spectrometry (LC-MS/MS) method was adopted to simultaneously detect the concentration of 25(OH)D3, 25(OH)D2, 3-Epi-25(OH)D3 by the QTRAP 5500 LC-MS/MS system (AB SCIEX Instruments, Framingham, MA, USA). Although 1,25(OH)D3 is the biologically active form of vitamin D, it may not be a reliable measure to quantify the vitamin D status because of its relatively short circulating half-life (4 to 6 h) when compared to that of 25(OH)D (2 to 3 weeks) [16]. The accuracy of the LC-MS/MS method for 25(OH)D measurement used in this study was verified against the samples from the Vitamin D External Quality Assessment Scheme, in which our results were within ±15% of the target value [17]. In this study, the total serum 25(OH)D concentration, which was defined as the sum of 25(OH)D3 and 25(OH)D2 minus 3-Epi-25(OH)D3, was used to reflect the vitamin D status. The total serum 25(OH)D concentration of less than 75 nmol/L was used as the cut-off level for vitamin D insufficiency, and thus the sample was categorised into two subgroups, namely the vitamin-D sufficient subgroup and the vitamin D-insufficient subgroup. This cut-off level was recommended in the clinical practice guidelines of the Endocrine Society Task Force on Vitamin D [18]. The serum level of various markers on mineral metabolism including albumin, ALP, calcium, phosphate, and ferritin was measured by a certified laboratory at a local public hospital.

#### 2.2.2. Caffeine Intake

Pregnant women were asked to report their frequency of food and beverage consumption in the past month prior to the dietary assessment using an electronic version of the Food Frequency Questionnaire (eFFQ) [19]. The eFFQ contains a total of 311 food items in twelve food groups including fish and seafood, mushrooms, eggs, dairy beverages, beans, fruits, grains, meats, snacks, soups, vegetables and condiments and oil [19]. Participants were asked to recall their intake of each food item in the preceding month. The level of caffeine intake was then calculated using Nutrition Analysis and Fitness software Food Processor (Version 11.6.1, ESHA Research, Salem, OR, USA) with reference to the USDA food composition table as well as the traditional Chinese and local Hong Kong food composition tables.

#### 2.2.3. Supplementation Intake

Pregnant women were asked to report whether they have taken any form of vitamin D-related supplement, including multivitamins and vitamin D, in their pregnancy. They were also asked to report their duration of vitamin D supplementation intake during pregnancy with options of “never”, “less than 1 month”, “1–4 months” and “5–9 months”.

### 2.3. Statistical Analyses

Statistical analyses were performed using the Statistical Package for the Social Sciences for Windows version 26.0 (IBM Corp, Armonk, NY, USA). All variables were examined for their distribution, outliers, and missing data before analysis. The assumption of a normal distribution was analysed by skewness and kurtosis. Potential outliers were removed if values were ≥3 standard deviations from the group mean. Descriptive statistics were computed to summarize the demographics, blood profile, and dietary intake pattern for vitamin D-sufficient and -insufficient pregnant women subgroups, respectively. The level of caffeine consumption and serum ferritin and ALP values were logarithmically transformed due to their skewed nature. A series of independent t-tests (for continuous variables) and chi-square analyses (for categorical variables) were conducted to detect differences in sociodemographic characteristics, blood profile, and dietary intake between the subgroups. To examine the associations between caffeine intake (independent variable) and various biochemical markers (dependent variables), three sets of regression models were built and tested in the vitamin D-sufficient subgroup and the vitamin D-insufficient subgroup, respectively. First, univariate analyses were conducted to examine the crude associations between caffeine intake and biochemical markers. In the second set, we adjusted the models for maternal age and family income level. The third set of models additionally adjusted for the average UV value during the month of collecting blood samples to minimize the potential effect of seasonality. All tests were two-tailed with *p* < 0.05 denoting statistical significance.

## 3. Results

This study recruited a total of 181 pregnant women with an average age of 32.9 years. Table 1 shows the demographics, blood profile, and dietary intake of pregnant women by their vitamin D status. Among 181 pregnant women, 50 (27.6%) of them were found to be vitamin D insufficient, and 131 (72.4%) were vitamin D sufficient. The sufficient and insufficient vitamin D subgroups did not differ by demographic characteristics, blood profile, and amount of caffeine dietary intake. As shown in Table 2a, results of univariate regression analyses showed an association between more caffeine intake and lower ALP levels within the sufficient vitamin D subgroup (β = −0.24, *p* = 0.006). After adjusting for maternal age, family income level, sun exposure intensity, and vitamin D supplementation duration, the association between caffeine intake and ALP level persisted. On the other hand, no associations were observed between caffeine intake and any of the biochemical markers within the insufficient vitamin D subgroup (Table 2b).

## 4. Discussion

This cross-sectional study examined the association between caffeine intake and mineral metabolism by the status of total serum 25(OH)D concentrations in pregnant women. Our study demonstrated an association between caffeine intake and ALP levels among vitamin D-sufficient pregnant women, even after adjusting for various confounding factors. Interestingly, such association was not observed in vitamin D-insufficient pregnant women. To the best of our knowledge, this study is the first to show that the health effects of caffeine intake during pregnancy may vary between women depending on their total serum 25(OH)D concentration, but this finding requires experimental research to confirm in future.

Our study found that among all the studied biochemical outcomes, only serum ALP levels had an association with caffeine intake. The ALP is an enzyme found in the bloodstream and many parts of the body including the liver, bones, and placenta of pregnant women [8]. The ALP has been used as a biomarker to indicate liver function and bone health. Elevated ALP level was found to be associated with increased risks of osteoporosis and liver disease [9,10]. Apart from bone and liver health, a previous literature review also found that higher serum ALP levels were associated with preterm delivery, hypertensive disorder, gestational diabetes, and low birth weight [20]. Elevated serum ALP levels are indeed common in pregnant women because of the resulting increase in placental and bone isoenzymes of ALP from maternal bone growth during this period [21]. However, there is an emerging body of evidence on the potential association between caffeine consumption and reduced serum ALP levels [22,23]. The National Health and Nutrition Examination Survey, for example, found a significant reduction in ALP levels for individuals who drink more than 3 cups of coffee per day [23]. Moreover, a Korean study found that current coffee drinkers had significantly lower ALP levels compared with never or past drinkers [24]. Our findings add to the current literature that caffeine consumption can also reduce the ALP levels of pregnant women when they have sufficient vitamin D.

While previous research has demonstrated that vitamin D sufficiency could mitigate adverse environmental effects [25], this study found a stronger effect of caffeine consumption on ALP levels among pregnant women with sufficient vitamin D compared to those without. Insufficient serum 25(OH)D concentrations may lead to bone and mineral homeostatic imbalance [26]. Since disrupting homeostasis is known to have many negative consequences, it is possible that the increase in serum ALP levels due to vitamin D insufficiency might offset its reduction due to caffeine consumption, thus resulting in minimal effects from caffeine intake among vitamin D-insufficient individuals. This is particularly plausible during pregnancy when the body undergoes constant changes [27]. However, the exact mechanism underlying the interplay between caffeine consumption, vitamin D status, and ALP level remains unclear and warrants further investigations.

Our results suggest that daily caffeine intake recommendations for pregnant women should be tailored to their health conditions. For example, stringent guidelines should be provided to unhealthy or vulnerable women, such as those with chronic medical conditions due to the potential adverse responses to caffeine intake. Healthy women should be alerted to the side effects of too much caffeine even though drinking coffee in moderation may not be harmful. Another possible approach is to encourage vitamin D-insufficient pregnant women to take vitamin D supplements regularly which can help promote the stability of physiological processes of the body. Future research should investigate the potential effect of the interplay between vitamin D supplementation and caffeine consumption on the bone health of pregnant women.

A key strength of this study was that the blood samples were collected in the period from the late second trimester to the early third trimester of pregnancy. This can better reveal the impact of caffeine consumption when the pregnancy is generally considered to be stable during this period. Another strength of this study was the use of eFFQ to conduct a comprehensive assessment of the total caffeine consumption during pregnancy. However, the findings of this study need to be interpreted with the following caveats. First, this was a cross-sectional study, and therefore, we were not able to establish a causal relationship between caffeine consumption and serum ALP levels. Second, our study only assessed the amount of caffeine intake at one time point, and thus we cannot draw a conclusion on the cumulative effect of caffeine consumption. Other unmeasured factors, such as supplementation dosage, weight changes, medication and therapeutic history, and use of other supplements might also relate to vitamin D level fluctuations during pregnancy and should be examined in future research. Third, our study cannot differentiate the specific types of ALP, such as placenta or liver/bone/kidney ALP, which might hinder the interpretation of our results, even though previous studies have reported associations of total serum ALP levels with other health outcomes of pregnant women and infants [28]. Lastly, the total 25(OH)D and ALP levels were measured at the same time point and thus we cannot infer the stability of vitamin D status and its impact on the association. In order to clarify the temporal relationship between caffeine consumption, ALP level, and vitamin D status during pregnancy, future work should conduct a longitudinal assessment of food and beverage consumption and measure 25(OH)D concentration and biochemical outcomes at multiple time points across the whole pregnancy period.

## 5. Conclusions

In conclusion, our study provides new insights into vitamin D status as a potential modifier of the health effects of caffeine consumption during pregnancy. Specifically, caffeine consumption was associated with a reduction in serum ALP levels among vitamin D-sufficient pregnant women, suggesting the potential benefits of caffeine for bone and liver health in pregnant women. Further studies are needed to confirm the long-term physiological effects of caffeine consumption during pregnancy and its underlying mechanism.

## Figures and Tables

**Table 1:** Demographics, blood profile, and dietary intake of pregnant women.

	Mothers with Sufficient Vitamin D Level (*n* = 131)	Mothers with Insufficient Vitamin D Level (*n* = 50)	Comparison *p*
Demographic characteristics			
Maternal age, mean (SD)	32.82 (3.74)	33.02 (0.59)	0.894
Family income, mean (SD)	53564.4 (33925.8)	52619.5 (33341.7)	0.779
Vitamin D supplementation duration, *n* (%)			0.122
Never	25 (19.08)	16 (32.00)	
Less than 1 month	13 (9.92)	1 (2.00)	
1–4 months	25 (19.08)	14 (28.00)	
5–9 months	61 (46.56)	17 (34.00)	
Whole pregnancy period	7 (5.34)	2 (4.00)	
Sun exposure average UV (blood collection month), mean (SD)	2.44 (0.34)	2.44 (0.34)	0.870
Biochemical outcomes			
Total 25(OH)D (nmol/L), median (IQR)	101.09 (28.22)	62.08 (10.60)	<0.001
Albumin (g/L), mean (SD)	30.91 (2.15)	30.80 (2.29)	0.723
Alkaline Phosphatase (IU/L), median (IQR)	66.00 (22.00)	64.66 (15.70)	0.878
Calcium (mmol/L), mean (SD)	2.21 (0.07)	2.20 (0.08)	0.298
Adjusted calcium (mmol/L), mean (SD)	2.28 (0.07)	2.26 (0.07)	0.288
Phosphate (mmol/L), mean (SD)	0.95 (0.12)	0.96 (0.13)	0.434
Ferritin (pmol/L), median (IQR)	65.00 (59.00)	43.00 (36.00)	0.100
Dietary intake			
Caffeine intake (mg/d), median (IQR)	6.89 (39.21)	10.46 (62.32)	0.553

Note: IQR: Interquartile range; SD: standard deviation.

**Table 2:** (**a**) Association between caffeine intake and biochemical outcomes in pregnant women with sufficient vitamin D; (**b**) Association between caffeine intake and biomedical outcomes in pregnant women with insufficient vitamin D.

**(a)**						
**Sufficient vitamin D**	**Model A**		**Model B**		**Model C**	
	**β**	***p***	**β**	***p***	**β**	***p***
Albumin	−0.04	0.692	−0.01	0.930	0.01	0.930
Alkaline Phosphatase	−0.24	0.006	−0.23	0.009	−0.24	0.006
Calcium	−0.03	0.770	0.003	0.977	0.00	0.985
Adjusted calcium	−0.02	0.821	0.00	0.999	−0.01	0.937
Phosphate	0.13	0.155	0.13	0.147	0.14	0.125
Ferritin	−0.05	0.611	−0.04	0.665	−0.02	0.826
**(b)**						
**Insufficient Vitamin D**	**Model A**		**Model B**		**Model C**	
	**β**	***p***	**β**	***p***	**β**	***p***
Albumin	0.05	0.741	−0.01	0.927	−0.01	0.935
Alkaline Phosphatase	0.04	0.811	−0.02	0.914	−0.02	0.917
Calcium	−0.03	0.833	−0.10	0.470	−0.10	0.479
Adjusted calcium	−0.07	0.646	−0.12	0.384	−0.12	0.392
Phosphate	0.02	0.883	−0.02	0.904	−0.02	0.917
Ferritin	0.03	0.863	0.01	0.963	0.01	0.963

Model B: Model A adjusted for maternal age and family income; Model C: Model B further adjusted for sun exposure average UV value.

## Data Availability

The data that support the findings of this study are available from the corresponding author upon reasonable request.
